# Focus on Mental Health During the Coronavirus (COVID-19) Pandemic: Applying Learnings from the Past Outbreaks

**DOI:** 10.7759/cureus.7405

**Published:** 2020-03-25

**Authors:** Kaushal Shah, Dhwani Kamrai, Hema Mekala, Birinder Mann, Krishna Desai, Rikinkumar S Patel

**Affiliations:** 1 Psychiatry, Griffin Memorial Hospital, Norman, USA; 2 Internal Medicine, Terna Medical College, Mumbai, IND

**Keywords:** covid-19, corona virus, 2019-ncov, pandemic, social and behavioral epidemiology, mental health services, behavioral problem

## Abstract

The 2019 novel coronavirus (COVID-19) has gained global attention after it originated from China at the end of 2019, and later turned into pandemic as it affected about 118,000 in 114 countries by March 11, 2020. By March 13, 2020, it was declared a national emergency in the United States as the number of COVID-19 cases, and the death toll rose exponentially. To contain the spread of the disease, the world scientist community came together. However, the unpreparedness of the nations, even with the advanced medical sciences and resources, has failed to address the mental health aspect amongst the public, as all efforts are focused on understanding the epidemiology, clinical features, transmission patterns, and management of COVID-19 pneumonia. Our efforts in this review are to evaluate and study similar outbreaks from the past to understand its adverse impact on mental health, implement adequate steps to tackle and provide a background to physicians and healthcare workers at the time of such outbreaks to apply psychological first aid.

## Introduction and background

An outbreak of a global pandemic causes fear and concern among many and reportedly influence the cognitive well-being of every individual. The lives of infected individuals, family and friends, and the society are at stake due to the perpetuated potential effects of the 2019 novel coronavirus (COVID-19). The outbreak that started in China turned into pandemic as it infected more than 118,000 people in over 114 countries by March 11, 2020 [1]. On March 17, 2020, the COVID-19 outbreak was declared a national emergency in the United States as the number of cases grew over 4,226 with a death toll of about 75 [2]. Globally, there are 105,586 infection cases reported as of March 8, 2020 with 3,656 new infections while cases reported in China alone of COVID-2019 pneumonia are 80, 859 [1]. The current death toll is at 3,584 globally of which 3,100 are in China as of March 8, 2020 [1].

There is a neuropsychiatric linkage between the outbreak of acute respiratory infections and mental disorders which date back to the prevalence of influenza and severe acute respiratory syndrome (SARS) that took place years ago. The people who are in quarantine areas may experience boredom, anger, and loneliness; the symptoms of the viral infection such as cough and fever may also cause worsening cognitive distress and anxiety among people due to the fear of contracting the COVID-19 [3]. During the early phase of the manifestation of SARS, several psychiatric comorbidities such as depression, panic attack, anxiety, psychomotor excitement, suicidality, delirium, and psychotic symptoms were reported [3].

Ever since the COVID-19 outbreak, scientists, clinicians, and health authorities across the globe are trying to build consensus on the clinical presentation and symptoms of COVID-19. A study assessing the clinical characteristics of COVID-19 pneumonia reveals that the most common symptoms of the infection are fever and cough. Among the studied 1,099 patients with the confirmed case of the disease in 30 provinces and 552 hospitals, 43% and 67% of them had a fever and cough on admission respectively, while 88.7% of them were hospitalized [4]. About 54% of the patients showed ground-class opacity as the most common radiologic finding, whereas 82% of the patients showed lymphocytopenia on admission [4]. This virus is also known to be transmitted by mildly ill or pre-symptomatic infected persons, which pose a challenge to control compared to the middle east respiratory syndrome (MERS) and SARS pandemics [5].

Among the confirmed 72,314 reported cases as of February 11, 2020, the most affected age group is 30-79 years, which had 38,680 cases of the 44,672 confirmed cases [6]. The least affected age groups are persons below 20 years of which among the reported, were a total of 965 cases as of February 11, 2020 [6]. The incubation period of the infection is between two and 27 days with a median of 14 days due to which the World Health Organization (WHO) and Centers for Disease Control and Prevention (CDC) recommend 14 quarantine days [1, 7]. The infection is believed to spread person to person primarily through droplets from the nose or mouth [1, 7].

While the scientific community and the WHO are still working on many unanswered aspects of this outbreak, clinicians and the general public are responding to this uncertain situation based on the limited confirmed information. This dubious situation has already created a large scale of disturbances in the lives of people across the globe, which calls for the need for research to study its implication on the mental health based on the learning of past outbreak.

Literature searches were carried out on PubMed, EMBASE, and Google Scholar using the keyword “COVID-19”, “coronavirus”, “2019-nCoV”, “pandemic,” and cross-referencing it with “mental health”, “behavioral problem”, “emotional distress,” and “psychological distress”. Abstracts found through this indexed search were further reviewed and screened to identify relevant articles or studies for our literature review. Our review article focused on 1. current issues and intervention to handle COVID-19 pandemic; 2. to understand the mental health impact on patients and at-risk population and the healthcare professionals; and 3. steps focusing on mental health and psychological first aid.

## Review

Current issues

The WHO and CDC, and other health authorities across the globe are currently focusing on containing the COVID-19 pneumonia pandemic by recommending measures such as social distancing and quarantine; however, it lacks the emphasis and intervention of its impact on mental health [1]. COVID-19 infection is a new disease; hence it is important to understand that its emergence and spread may lead to cognitive distress, anxiety, and fear in the public which then may lead to harmful stereotypes [1]. With rising public stigma can cause the affected individuals hiding their illness to avoid discrimination which may prevent them from seeking immediate healthcare intervention [1].

The implementation of home quarantine is the number one factor that increases the prevalence of medical practitioners developing brief/acute to post-traumatic stress disorder (PTSD) as they display increased sleep and numbness disorder [8]. The total number of infected health personnel as of February 11, 2020 was 1,716 (3.8%) of the total confirmed 44,672 cases with five reported deaths [6]. Notably, there are not enough services that are set up to provide psychological counseling and psychiatric screening services for anxiety, depression, and suicidality for medical practitioners dealing with infected patients [9]. 

The counteractive measures employed by the health authorities across the world towards managing the COVID-19 infection include: early identification and separation of suspected cases, tracing of contacts, biological and clinical data collection from patients national and regional criteria for diagnosis, the consensus of expert medical interventions, hospitals and units established for isolation and the prompt increase in the number of medical practitioners to the affected regions [10]. These intervention measures are focusing on combating the pandemic but have serious mental health effects on the medical teams and the population at large [11].

Synopsis of current interventions

The prominent health authorities across the world have provided recommendations for the benefit of public health. The WHO advised people to follow social distancing and avoid close contact with anyone, especially from the person showcasing any respiratory symptoms [12]. They also emphasized on maintaining better hygiene through washing hands consistently and use of appropriate protective gears while using dealing with wild and farm animals [12]. As the number of people visiting the hospitals and clinics is rising, they have advised the EDs and health centers to enhance the standards of infection prevention and control practices, and also directed to practice hygiene etiquette [12].

In addition to the above measures, the CDC recommended healthcare organizations to focus on the needs of medical care and assess the required resources. The objective is to ensure the preparedness of the healthcare system by outreaching professionals and clinical organizations. The readiness is measured in terms of the availability of resources to ensure a sufficient amount of emergency funds, the number of guidance documents for the public to educate about infection and control measures, medical supplies, personal protective equipment, and clinical management. With the collaborative approach, they are working closely with healthcare and nonhealthcare organizations, public health departments, pharmaceutical companies, and religious organizations of all the states to reduce the spread of the COVID-19 infection [13]. As the current focus of the health authorities is mainly on the prevention, management, and limiting the spread of the coronavirus infection, a little attention is on mental health. No specific interventions are provided and executed to protect the mental health in the community, including healthcare workers [14]. 

Mental health impact on patients and the general population

A study was done to assess the psychological and immediate stress outcomes on patients who were quarantined and put under hemodialysis and the medical practitioners who cared for the infected persons at the time of the MERS [15]. With the help of mini international neuropsychiatric interview technique and utilizing the hospital anxiety and depression scale, it was determined that the patients had higher impact events scale-revised scores (IES-R) during the initial phase of the outbreak [15]. Qualitatively, an assessment of the high-risk groups showed varied IES-R scores on sleep and numbness; this was dependent on the application of home quarantines [15]. The results are consistent with the study on the Ebola virus's impact on the affected individuals in Nigeria that aimed at examining the psychological distress of the survivors [16].

A study detailing the psychological trauma of bereaved families and victims of MERS states that a family affected by the infection claimed that the general public avoided them, and were socially isolated even after being treated and declared free of the disease [17]. There are also perpetuations about incubation periods of the infection being considered longer than usual by the public due to mixed information from electronic sources; and with a higher level of uncertainty, rumors get exaggerated [17]. The case of COVID-2019 is not different compared with the MERS and SARS cases as there are similar claims on social media platforms about the severity of the infection which infiltrates fear and worries among the public with increased anxiety levels [18]. In a study done on the terminal psychiatric disorders among the survivors of SARS, it was revealed that 25% of the patients showed signs of PTSD while 15.6% of them had worsening depression [19]. This correlates to the high number of older adult suicide deaths witnessed in Hong Kong in 2003 and 2004 among the affected individuals during the SARS epidemic [20]. MERS survivors of the critical illness also reported a low quality of life than those indirectly affected [21].

Mental health impact on healthcare workers

Previous studies on SARS and Ebola reveal a severity in emotional distress during the outbreaks of such epidemics [15]. It is also worthwhile to presume that many medical practitioners face PTSD, depression, anxiety, and burnout after the cessation of the incidence of such infections [15]. Compared with the number of other healthcare professionals who participated in the study, the clinicians showed a higher intrusion sub-score than those that never took part [15]. The results are consistent with studies on the SARS outbreak which demonstrated that 18%-57% of medical providers experiencing emotional distress at the onset, during, and after the outbreak of the infection [22]. During the Ebola outbreak, many health workers without traditional patient care roles were mostly infected; in addition, the medical staff worked extra-hours and settings without personal protective equipment and driven mainly by compassion [23]. The situation with COVID-19 is not different and poses a more significant mental health effect on the medical practitioners, as witnessed in the case of one medical practitioner dying due to the infection as purported [24].

A study found that hardiness and stigma have both direct and mediated impacts through stress among nurses working in government hospitals during the outbreak of MERS due to deteriorated mental health [25]. Clinicians have profound psychological distress due to the SARS epidemic than the nurses; this brings in the line of opinion that different professional levels have a disparate mental health impact [26-27]. A different study reaffirms this assumption in that the Davidson trauma scale (Chinese version) helped reveal a significant difference in the severity of PTSD in the ED than the clinicians in the psychiatric wards [28]. 

Learning from the past outbreaks

According to the Institute of Medicine, during the SARS outbreak of 2002-2003, four ethical issues were raised: the roles and responsibility of the medical workers, the impact of the infection on the global economy, equitable care, and the challenge of balancing public welfare and individual rights [29]. The issues saw a new era of international public health [29]. The emergence of internationally spreading epidemics reduces economic activities; thus, governments have the challenge of preventing the spread of the infection while minimizing the economic effects of the pandemic on travel and trade restrictions [29]. The outbreak of pandemics has a potential impact on the existing illnesses, causes distress among caretakers, and affected persons and leads to an onset of mental symptoms among the young or old, which is possibly related to the interplay of mental disorders and immunity [30]. In order to avoid the mental health effects of the COVID-19 infection, people need to avoid excessive exposure to COVID-19 media coverages, maintain a healthy diet and positive lifestyle, and reach out to others for comfort and consolation that the situation will soon be contained. Everyone should maintain a sense of positive thinking and hope and take personal or group time to unwind and remind the self that the intense feelings of fear, panic, and anxiety will fade. Additionally, seek information from reputable government sources for information and avoid the spread of erroneous information on the internet.

The previous outbreaks of the influenza pandemic have led to the incorporation of a resilience training program for medical practitioners in the preparation of another pandemic [31]. The training was also seen as a way of protecting the capacity of the medical institutions in responding to epidemics or pandemics. This strategy, just as the current case of COVID-19 and other infectious disease outbreak requires the following themes of concern in learning from the past epidemics or pandemics [31]: 1. balance of family and work; 2. ensuring reliability, consistency, and time-bound information; 3. education and preparation of the employees and the involved communities; 4. ensuring fairness and addressing ethical concerns; 5. front-end leaders’ participation; 6. validating and valuing the front-line staff’s contribution; 7. timely addressing of fears and worries among the medical team; and 8. ardent information on medical staff's redeployment to high-risk areas.

Steps focusing on mental health

Psychiatric treatment team including nursing staff, psychiatrists, case managers, and psychologists and social worker should be established to deliver mental health support to the affected persons and medics [3]. This should be coupled with the creation of appropriate mental health services, facilities, and specialized psychiatric treatment for patients with comorbid cognitive disorders.

Clear and consistent information should be provided to the medical teams on the prevalence of the COVID-19, the charted plans for treatment, the progress, and the updates on the status of health should be provided to both the patients and families involved [3].

The government and health organizations should ensure secure electronic information-sharing platforms are used to provide and promote telepsychiatry and telemedicine psychological counseling, promote legal information, and eliminate cases of isolation [32]. There should be more enforcement on the awareness of online training in the management of COVID-19 [33].

Time-bound behavioral therapy should be provided to persons exhibiting signs of mental disorders to reduce the cognitive effects of the pandemic [34]. The psychiatrists should also allow for personal adjustment to face the situation; this involves the behavioral and emotional responses, which, when coupled with the psychotherapeutic treatments based on the model of stress adaptation [3].

There should be more research and clinical trials to come up with effective antiviral prophylaxis treatment of the infectious disease, as seen in the previous pandemics of SARS, MERS, and Ebola [35].

Importance of psychological first aid

Psychological first aid (PFA) is a crucial early intervention that focuses on mental health of the affected survivors by providing psychosocial support during outbreaks like COVID-19. It is a tool designed to mitigate acute distress and assess the need for continued mental healthcare through compassionate and supportive presence [36]. Relative to the previous pandemics, the COVID-19 has an impact on mental health either directly or indirectly [37]. PFA is essential to bridge the collaborative services and coping information among COVID-19 affected individuals [37].

Several PFA frameworks and models are currently available for emergency management. The Johns Hopkin’s PFA tool consists of the 'RAPID' model with five steps [38]: step one, rapport and reflective listening: used throughout the interaction with a person in crisis, and the goal is to make initial contact and establish rapport through active and reflective listening techniques such as paraphrasing with empathy; step two, assessment: focuses on the evaluation of psychological and basic physical needs; step three, prioritization: emphasizes the importance of triage principles by focusing on the severity of cases that needs emergent care; step four, intervention: aims to mitigate distress and try to functional capacity using cognitive and behavioral interventions, and also provide basic needs; and step five, disposition and follow-up: the final step is a continuous process until stabilization of the situation, through providing constant support, meeting their needs, and regular monitoring.

Fundamentally, all tools aim to calm and orient emotionally overwhelmed survivors, offer practical help, contact, and engage, provide safety and comfort, and gather information on the present concerns and needs [37]. Thus, the PFA is an essential tool for clinicians and the survivors in addressing stress-related reactions after traumatic events like the COVID-19 pandemic [37].

## Conclusions

Substantial evidence from the past studies of the impact of SARS, MERS, influenza, and Ebola epidemics on the at-risk population, the suffering individuals and healthcare providers showed neuropsychiatric linkage. The results are relative to the current COVID-19 pandemic; they infiltrate fear, anxiety, emotional distress, and post-trauma stress symptoms as the affected individuals are viewed as minority and secluded from the rest of the population. The intervention measures that are employed by various health authorities and government bodies in combating the infection may help in eliminating the threat during the time of uncertainty; however, the multivariate studies done on the previous outbreaks show that they have long-term cognitive and mental health effects on the population. It is vital to emphasize the mental health well-being of the population and take proactive steps to minimize its detrimental effects during the COVID-19 pandemic.
